# Sparing spiders: faeces as a non-invasive source of DNA

**DOI:** 10.1186/s12983-015-0096-y

**Published:** 2015-01-28

**Authors:** Daniela Sint, Isabella Thurner, Ruediger Kaufmann, Michael Traugott

**Affiliations:** Institute of Ecology, University of Innsbruck, Technikerstraße 25, 6020 Innsbruck, Austria

**Keywords:** Lycosidae, Molecular species identification, Molecular prey detection, Multiplex PCR

## Abstract

**Introduction:**

Spiders are important arthropod predators in many terrestrial ecosystems, and molecular tools have boosted our ability to investigate this taxon, which can be difficult to study with conventional methods. Nonetheless, it has typically been necessary to kill spiders to obtain their DNA for molecular applications, especially when studying their diet.

**Results:**

We successfully tested the novel approach of employing spider faeces as a non-invasive source of DNA for species identification and diet analysis. Although the overall concentration of DNA in the samples was very low, consumer DNA, suitable for species identification, was amplified from 84% of the faecal pellets collected from lycosid spiders. Moreover, the most important prey types detected in the gut content of the lycosids were also amplified from the faecal samples.

**Conclusion:**

The ability to amplify DNA from spider faeces with specific and general primers suggests that this sample type can be used for diagnostic PCR and sequence-based species and prey identification such as DNA barcoding and next generation sequencing, respectively. These findings demonstrate that faeces provide a non-invasive alternative to full-body DNA extracts for molecular studies on spiders when killing or injuring the animal is not an option.

**Electronic supplementary material:**

The online version of this article (doi:10.1186/s12983-015-0096-y) contains supplementary material, which is available to authorized users.

## Introduction

Spiders are a diverse invertebrate group with more than 37,000 described species [1]; they inhabit almost all terrestrial ecosystems, where they are important arthropod predators [2-4]. Identifying spiders based on their morphological traits can be challenging (especially in the case of juveniles and females), but DNA-based identification can simplify this task [5,6]. Similarly, as spiders are liquid feeders, morphological identification of prey remains is of limited use in studying their feeding ecology. Again, molecular tools significantly improved the diet analysis of spiders: solid prey remains (e.g. from webs) are no longer needed to analyse what has been consumed [7-9]. The flip side of the coin is that both molecular identification and prey detection usually requires killing the animals to dissect their gut or using the whole animal for DNA extraction. For species identification, it is at minimum necessary to injure the spider when inducing autotomy of legs [10]. This is because, with few exceptions, only juveniles will moult and be able to replace missing limbs [2]. This renders these techniques unsuitable for situations in which spider identity and/or prey need to be determined non-invasively, e.g. when investigating threatened species or to identify spiders for subsequent ethological studies. Different types of non-invasively collected samples have been successfully used for molecular analysis, but most of these assays have been developed for vertebrates, which more readily provide suitable sample types such as hairs, feathers, saliva, regurgitates, shed skin, and faeces [11]. Less work has been conducted on invertebrates: slugs were identified based on body swabs [12], high-quality prey DNA was retrieved from carabid regurgitates [13,14], and DNA was successfully analysed from faeces of lobsters [15], millipedes [16], and beetle larvae [17]. In spiders, the complete exuviae of large tarantulas provided enough DNA for molecular species identification [18], but mature spiders usually do not moult [2], making it is impossible to obtain this sample type from adult individuals. Even if an individual will continue moulting, it might be necessary to keep that spider for a lengthier time until the skin is shed. Spiders do not provide regurgitates or mucus, which proved to be a useful source of DNA in other invertebrates. Faeces may provide a valuable source for non-invasively collected DNA samples, but the suitability of spider faeces for molecular analysis has never been tested.

Here we explore whether spider faeces enable non-invasive species identification and diet analysis. Field-collected wolf spiders (Lycosidae) and the corresponding faecal material produced upon collection were used to compare the success in molecular prey detection and species identification between the two sample types (full-body DNA extract and faecal sample) using a series of multiplex PCR assays. This effort included the application of two newly developed PCR systems.

## Results

In all 189 full-body DNA extracts of wolf spiders, DNA of *Pardosa* spp. was detected at genus and at species level using the multiplex PCR systems IPC [19] (detects intraguild predation and collembolan DNA) and DUP (duplex PCR system detecting DNA of *P. nigra* and *P. saturatior*), respectively. A total of 185 faecal samples (98%) contained amplifiable DNA: these were compared to the respective full-body DNA extracts regarding their suitability for molecular analysis. Overall the detection of lycosid DNA was possible in 84% of the 185 faecal samples: 73% of the faecal samples tested positive at genus level (IPC assay) and 72% scored positive at species level for DNA of *P. saturatior* or *P. nigra* (DUP assay). In 95% of the 130 cases in which both the faecal and full-body sample could be assigned to one of the two *Pardosa* species, the identification was congruent between the two sample types. In five out of the six divergent samples, DNA of both *Pardosa* species was found in the full-body DNA extract (see below).

In 22 of the full-body samples and in one faecal sample, DNA of both *Pardosa* species was detected by DUP. This indicates predation among the two species. In all but two of these samples, one of the two amplicons showed at least double the signal strength compared to the other one (mean signal ratio strong : weak was 17:1). This enabled differentiating the consumer (strong signal) from the prey (weak signal). Taking this differentiation into account, 99% of the lycosids could be identified from the full-body DNA extracts as *P. nigra* (146 individuals) and P*. saturatior* (37 individuals).

**Figure 1:**
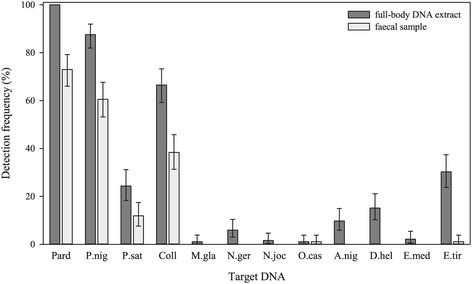
**Detection frequencies of consumer- and prey-DNA.** Detection frequencies of lycosid and prey DNA in DNA extracts of full-body and faecal samples of lycosid spiders collected in three glacier forelands using three diagnostic multiplex PCR assays. Targeted DNA: *Pardosa* spp. at genus level (Pard), *P. nigra* (P.nig), *P. saturatior* (P.sat), Collembola (Coll), glacier harvestman *Mitopus glacialis* (M.gla), carabid beetles [*Nebria germari* (N.ger), *N. jockischii* (N.joc), *Oreonebria castanea* (O.cas)], and linyphiid spiders [*Agyneta nigripes* (A.nig), *Diplocephalus helleri* (D.hel), *Entelecara media* (E.med), *Erigone tirolensis* (E.tir)]. DNA of the linyphiid *Janetschekia monodon* and of the carabid *N. rufescens* were not detected. Error bars represent 95% tilting confidence intervals from 9999 bootstrap resamples; non-overlapping confidence intervals are interpreted as significant differences.

When testing the full-body extracts for prey DNA using the LIN (PCR system detecting DNA of five linyphiid species) and IPC assays, nine non-*Pardosa* prey taxa were detected: DNA of collembolans and the linyphiid *Erigone tirolensis* were amplified in 123 and 56 samples, respectively. This was significantly more often than the many other prey tested for (Figure 1). The number of individuals where at least one prey type was detected was significantly higher in the full-body (79%) compared to faecal samples (39%; χ^2^ = 61.32, p < 0.001). Overall, only three non-*Pardosa* prey taxa could be detected in the faecal samples, whereby DNA of collembolans was detected most frequently (38%) and two faecal samples each tested positive for DNA of *E. tirolensis* and *Oreonebria castanea* (Figure 1).

## Discussion

This study demonstrates that spider faeces can be used for both species identification and prey detection, which enables a non-invasive examination of spiders. Although mainly genus- and species-specific primers were applied in this study to identify the consumer and its prey, DNA from spider faeces was also successfully amplified with general primers which are commonly used for DNA barcoding species and for sequence-based prey identification (Additional file 1; [20,21]). This demonstrates that the suite of molecular techniques that can be employed to analyse DNA from spider faeces is not limited to highly-specific diagnostic PCR assays, but that other molecular methods for species and/or prey identification such as DNA barcoding approaches and next generation sequencing could potentially be applicable to this non-invasively derived sample type.

The ability to molecularly identify the consumer and the food from the same sample is important in ecological studies. We found that faeces were especially useful for species identification: consumer DNA was amplified from 84% of the samples. This percentage ranges on the higher end of the scale for the presence of consumer DNA in non-invasive samples reported from other animals (e.g. [16,22-24]). Molecularly differentiating full-body DNA extracts between *P. nigra* and *P. saturatior* by the newly-developed duplex PCR system, which was optimized to amplify both species with approximately the same efficacy, enabled us to identify whether DNA of *P. nigra* or *P. saturatior* dominated the sample [19]. As full-body DNA extracts contain consumer DNA in large excess over prey DNA [25], we could identify predator and prey also in samples where both types of DNA were present. The few discrepancies in species identification between full-body and faecal samples probably reflect the absence of consumer DNA in the faeces, i.e. only the DNA of the consumed wolf spider was present and misidentified as indicating the consumer. This interpretation is supported by the fact that in five out of six questionable samples, DNA of both *Pardosa* species was present in the full-body DNA extracts. In one wolf spider, DNA of *P. nigra* and *P. saturatior* was even present in both sample types, but the identification of the predator and prey, based on amplicon strength, was not congruent (the difference was clear for the full-body DNA extract, but in the faeces the prey gave a slightly stronger signal). Nevertheless, this ability to identify a spider based on its faeces is an encouraging result for further investigations, as for example when living spiders need to be identified for field or laboratory studies.

Although prey DNA detection success was significantly lower in faeces than in full-body samples, the most common prey items were also detected in the non-invasive samples. The reduced ability to detect prey DNA in faeces is probably due to the high efficiency with which spiders digest their food [2]. This yields a very low DNA content in the faecal samples, which was <1 ng/μl in the current study (see Additional file 1). Nonetheless, using spider faeces as a source of dietary information is useful to get a general idea of the spider diet in situations where the whole spider cannot be killed to obtain a full-body DNA extract to perform molecular gut content analysis. Such situations arise, for example, when prey choice needs to be examined in rare and/or protected spiders, in habitats such as in national parks where killing animals is prohibited, or when the impact of lethal sampling on the studied system needs to be minimized.

We expected spider faeces to have a DNA content comparable to beetle regurgitates, typically containing between 1 and 10 ng ds DNA/μl [26], and which are well suited to track the beetle’s diet [13,14]. In the present faecal samples, however, the observed DNA content was much lower (<1 ng ds DNA/μl). We therefore suggest optimizing the DNA extraction process to increase the DNA concentration and, consequently, the robustness to detect and identify prey DNA. One strategy to achieve a higher DNA concentration would be to reduce the volume of buffer when eluting (silica-based extraction protocols) or resolving (e.g. CTAB protocols) the DNA at the end of the DNA extraction process. A concentrating step following DNA extraction could also be performed. Another approach to increase the concentration of prey DNA in faecal samples would be to pool several faecal pellets of one individual into one sample for DNA extraction.

Our work indicates that obtaining a reasonable number of faecal samples is straightforward because more than 50% of the captured lycosid spiders defecated within a few hours upon collection. The number of faecal pellets that can be gathered after spider collection might be increased even further by feeding the animals, as many species defecate stored excrements when provided with fresh prey [2]. Prey DNA can be tracked in spiders for extended times post-feeding [27-29]: for example, cricket DNA could be detected in full-body DNA extracts of *Pardosa* spp. for a minimum of 84 h after a single meal [30]. It is likely that, along with small amounts of predator DNA, also prey DNA is continuously excreted during these long digestion times. While a single faecal pellet might not contain enough DNA to enable successful detection of prey and/or consumer DNA, the amount contained in several pellets might be sufficient. For prey detection it is important that the spider is not fed during the timespan in which faeces are collected to avoid mixing laboratory- and field-consumed prey. Alternatively, if feeding the spider is desired to enhance faecal production (see above), the spider can be fed with prey which does not occur in the habitat the spider was collected from. These strategies, together with the application of highly sensitive PCR assays or a next generation sequencing approach [31] – where the sequence information of individual molecules is read – might further improve the molecular information that can be obtained from spider faeces beyond the possibilities reported in this study.

## Methods

*Pardosa nigra* and *P. saturatior* (Araneae: Lycosidae) were collected by dry pitfall-trapping between 8 and 23 July 2010 in three neighbouring glacier forelands (Gaisbergtal, Rotmoostal, Langtal) in Tyrol, Austria. Spiders were placed individually in 2 ml reaction tubes, transported to a field station in a cool box, and frozen at −24 °C. In case defecation occurred during transport, the spider was transferred into a clean tube before freezing, and lysis-buffer was added to the tube containing the faeces. In total, 189 faecal pellets were obtained from 347 sampled spiders. For details on sample processing and DNA extraction see Additional file 1.

**Figure 2:**
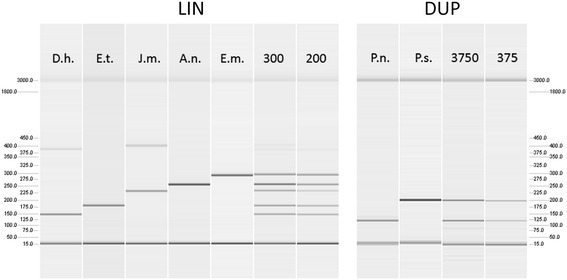
**Gel image of the newly developed multiplex PCR systems.** QIAxcel gel image of PCR products generated with the multiplex PCR system LIN and the duplex PCR system DUP. LIN: *Diplocephalus helleri* (D.h.; 151 bp), *Erigone tirolensis* (E.t.; 186 bp), *Janetschekia monodon* (J.m.; 240 bp), *Agyneta nigripes* (A.n.; 264 bp), *Entelecara media* (E.m.; 298 bp), artificial mixes containing 300 and 200 double stranded (ds) templates per target, respectively. DUP: *Pardosa nigra* (P.n.; 118 bp), *Pardosa saturatior* (P.s.; 202 bp), artificial mixes containing 3750 and 375 ds templates per target species, respectively. An internal marker is run alongside each sample (15 and 3000 bp), and the scale on the left and right side enables an estimation of fragment length. At higher template DNA concentrations, D.h. and J.m. may produce an additional amplicon of ~390 and ~400 bp, respectively.

Two new multiplex PCR systems (DUP, LIN) were developed (see Additional file 1) and, together with an already existing multiplex PCR system (IPC, [19]), used to screen the DNA extracts of the spiders and their faecal samples for consumer- and prey-DNA. First, the duplex PCR system (DUP, Figure 2) was applied to molecularly identify the samples as either *P. nigra* or *P. saturatior.* Second, all samples were screened for DNA of potential prey taxa common in the three glacier forelands using the LIN and IPC multiplex PCR systems: the LIN system (Figure 2) enables tracking of predation on five linyphiid spider species commonly occurring in the glacier forelands [6], while the IPC system detects predation on four species of carabid beetles (*Nebria germari*, *N. jockischii*, *N. rufescens*, *Oreonebria castanea*), the glacier harvestman *Mitopus glacialis,* and collembolans (IPC, [19]). Additionally, IPC amplifies DNA of *Pardosa* spp. as an internal positive control. PCR products were separated, visualized, and scored on QIAxcel (Qiagen, Hilden, Germany), an automatic capillary electrophoresis system.

To compare the detection frequencies of the different targets, 9999 bootstrap resamples were drawn with replacement from the observed data using the software TIBCO Spotfire S+ 8.1. Non-overlapping 95% tilting confidence intervals were used as a conservative estimation of significant differences.

## Additional file

Additional file 1:
**Details on sample processing and multiplex PCR systems.**
