# Supplementary material for: Sparing spiders: faeces as a non-invasive source of DNA
Source: Front Zool. 2015 Jan 28;12:3. doi: 10.1186/s12983-015-0096-y (PMC4341883; doi:10.1186/s12983-015-0096-y)
Supplement: Additional file 1: — Details on sample processing and multiplex PCR systems. [file 12983_2015_96_MOESM1_ESM.pdf]

Sample processing and DNA extraction

In the lab, collected lycosids were recorded, numbered, and every second spider was frozen for molecular gut content analysis. In case a spider had defecated into the reaction tube during transport, that spider was transferred into a clean reaction tube before freezing, and 440 µl lysis-buffer (TES buffer and proteinase K [1]) was added to the tube containing the faeces. All samples were stored frozen until DNA extraction.

Lycosid spiders were homogenized in 440 µl of TES buffer with 5-6 glass balls (diameter approx. 2 mm) in a Precellys 24 tissue homogenizer (PEQLAB, Erlangen, Germany). After overnight incubation at 56 °C, DNA extraction followed a modified CTAB protocol [1] with subsequent clean-up with the QIAquick PCR Purification Kit (Qiagen, Hilden, Germany) following the recommendations of the manufacturer.

Faecal samples were incubated at 56 °C for 3 h. Then the DNA was extracted from 200 µl of the lysate with a BioSprint 96 workstation (Qiagen) using the BioSprint 96 DNA Blood Kit (Qiagen). The extraction followed the manufacturer's recommendations, except that buffer ATL (Qiagen) was replaced by TES-buffer for lysis (see above) and that DNA was eluted in 1x TE buffer instead of buffer AE (Qiagen).

Extraction negative controls were run along with all samples to check for carry-over DNA contamination. Those controls were handled identically as the samples, but did not contain any sample material. Controls were checked with the different multiplex PCR systems to detect any potentially diverted DNA – none was found.

Multiplex PCR development

Primers targeting DNA of *P. nigra* and *P. saturatior*, respectively, were designed based on available sequences of the cytochrome c oxidase subunit one gene (COI) of high alpine species [2, 3]. Out of a larger set of primers developed for species identification of linyphiid and theridiid spiders in glacier forelands [3], primer pairs targeting five common European species of Linyphiidae (*Diplocephalus helleri*, *Entelecara media*, *Erigone tirolensis*, *Janetschekia monodon*, *Agyneta nigripes*) were selected and combined with a new reverse primer for *E. media* to a multiplex system (LIN). This new multiplex PCR system amplifies only DNA fragments shorter than 300 bp length and is thus suitable to detect also semi-digested DNA [4]. Primer concentrations for each multiplex PCR system were adjusted on the basis of standardized DNA templates [2], so that all targets were amplified at approximately the same efficiency. This yielded detection signals of comparable strength if the same amount of template DNA is present during PCR. The sensitivity of both optimized multiplex systems was determined with and without the addition of approx. 300 ng of non-target DNA (*Mitopus glacialis*) using standardized numbers of DNA templates. For the *Pardosa* duplex it was also verified that with 20-times excess of DNA of one species the other species is still detectable. The specificity of the multiplex PCR systems was tested with 53 different target and non-target taxa found/collected in high alpine environments. A complete list of all tested taxa and their corresponding amplification success is available in Table A1.

Table A1: List of non-target taxa tested with the different multiplex PCR systems (LIN and DUP). n – number of tested individuals; 0 – no amplification; T – amplification of target fragment

				LIN					DUP	
			n	*D. helleri*	*E. tirolensis*	*E. media*	*A. nigripes*	*J. monodon*	*P. nigra*	*P. saturatior*
Arachnida										
	Acari		3	0	0	0	0	0	0	0
	Linyphiidae									
		Agyneta nigripes	2	0	0	0	T	0	0	0
		Diplocephalus helleri	2	T	0	0	0	0	0	0
		Entelecara media	2	0	0	T	0	0	0	0
		Erigone atra	2	0	0	0	0	0	0	0
		Erigone tirolensis	2	0	T	0	0	0	0	0
		Janetschekia monodon	2	0	0	0	0	T	0	0
		Mecynargus paetulus	2	0	0	0	0	0	0	0
	Lycosidae									
		Pardosa giebeli	2	0	0	0	0	0	0	0
		Pardosa nigra	2	0	0	0	0	0	T	0
		Pardosa saturatior	2	0	0	0	0	0	0	T
	Opiliones									
		Mitopus glacialis	2	0	0	0	0	0	0	0
		Mitopus morio	2	0	0	0	0	0	0	0
Gastropoda			2	0	0	0	0	0	0	0
Insecta										
	Carabidae									
		Nebria germari	2	0	0	0	0	0	0	0
		Nebria jockischii	2	0	0	0	0	0	0	0
		Nebria rufescens	2	0	0	0	0	0	0	0
		Oreonebria castanea	2	0	0	0	0	0	0	0
	Diptera									
		Acalyptratae indet.	1	0	0	0	0	0	0	0
		Agromyzidae	3	0	0	0	0	0	0	0
		Anthomyiidae	4	0	0	0	0	0	0	0
		Bibionidae	3	0	0	0	0	0	0	0
		Calliphoridae	2	0	0	0	0	0	0	0
		Cecidomyiidae	1	0	0	0	0	0	0	0
		Chironomidae	6	0	0	0	0	0	0	0
		Chloropidae	2	0	0	0	0	0	0	0
		Dixidae	1	0	0	0	0	0	0	0
		Dolichopodidae	1	0	0	0	0	0	0	0
		Drosophilidae	2	0	0	0	0	0	0	0
		Empididae	1	0	0	0	0	0	0	0
		cf Heleomyzidae	1	0	0	0	0	0	0	0
		Muscidae	7	0	0	0	0	0	0	0
		Mycetophilidae	1	0	0	0	0	0	0	0
		Phoridae	6	0	0	0	0	0	0	0
		Psilidae	3	0	0	0	0	0	0	0
		Rhagionidae	1	0	0	0	0	0	0	0
		Sciaridae	5	0	0	0	0	0	0	0
		Simuliidae	1	0	0	0	0	0	0	0
		Sphäroceridae	1	0	0	0	0	0	0	0
		Syrphidae	3	0	0	0	0	0	0	0
		Tabanidae	1	0	0	0	0	0	0	0
		Tachinidae	2	0	0	0	0	0	0	0
		Tephritidae	2	0	0	0	0	0	0	0
		cf Therevidae	1	0	0	0	0	0	0	0
		Tipulidae	3	0	0	0	0	0	0	0
	Coleoptera spp.		7	0	0	0	0	0	0	0
	Collembola		4	0	0	0	0	0	0	0
	Cinara sp.		2	0	0	0	0	0	0	0
	Hymenoptera		9	0	0	0	0	0	0	0
	Lepidoptera		6	0	0	0	0	0	0	0
	Planipennia		2	0	0	0	0	0	0	0
	Sminthuridae		1	0	0	0	0	0	0	0
	Trichoptera		1	0	0	0	0	0	0	0

Duplex PCR system for *Pardosa* (DUP)

The optimized reactions were performed using the Multiplex PCR Kit (Qiagen) in a volume of 10 µl and contained 1.5 µl DNA extract, each primer at its corresponding concentration (Table A2), 1x Multiplex reaction mix, 5 µg bovine serum albumin (BSA), 0.25x Q-Solution and PCR grade water to adjust the volume. The cycling conditions on an Eppendorf Mastercycler (Eppendorf, Hamburg, Germany) included an initial activation step of 15 min at 95 °C, 35 cycles of 30 s at 94 °C, 3 min at 60 °C, 1 min at 72 °C, and final elongation for 10 min at 72 °C. The two primer pairs generate a DNA fragment of 118 bp length from *P. nigra* DNA and a 202 bp fragment from *P. saturatior* DNA and are easily distinguishable with QIAxcel (QIAGEN), an automatic capillary electrophoresis system, or with other electrophoretic systems (Figure 2 main text). The duplex PCR system is highly sensitive: 30 double-stranded templates were sufficient for both species to result in a detectable amplification of the target DNA. The addition of non-target DNA of *Mitopus glacialis* reduced the sensitivity to some extent so that 60 double-stranded templates were necessary to amplify a PCR product resulting in ≥0.1 relative fluorescent units (RFU) on QIAxcel. Both species can be detected with DNA of the other species present at a 20 times higher concentration.

Multiplex PCR system for linyphiid prey (LIN)

For the multiplex PCR system targeting five species of linyphiid spiders, the primer sequences, corresponding fragment length, and primer concentrations in the PCR are given in Table A2. Each 10 µl reaction contained 1.5 µl DNA extract, each primer at its corresponding concentration (Table A2), 1x Multiplex reaction mix (QIAGEN), 5 µg BSA, 30 mM tetramethylammonium chloride (TMAC), and PCR grade water to adjust the volume. The optimized cycling conditions included an initial activation step of 15 min at 95 °C, 35 cycles of 30 s at 94 °C, 3 min at 62.5 °C, 1 min at 72 °C, and final elongation for 10 min at 72 °C. All diagnostic DNA fragments of the new multiplex system can be well separated with QIAxcel (QIAGEN) (Figure 2 main text) and are shorter than 300 bp, making them suitable to detect semi-digested DNA [4]. The multiplex PCR assay is highly sensitive: 60 double-stranded template molecules were sufficient to produce amplicons with a signal strength ≥0.1 RFU

for both single species tested or an equal mixture of all species. For most species, 30 template molecules were also sufficient; only the signal strength of *J. monodon* dropped below 0.1 RFU. Detection limits were not negatively influenced by the presence of a high amount of non-target DNA. If a higher amount of target DNA was present, *D. helleri* and *J. monodon* might result in an additional weaker band of ~390 and ~400 bp lengths, respectively. As those fragments are considerably longer than the longest diagnostic fragment included in the multiplex system (298 bp), they can be well distinguished from target bands.

Table A2: Primer pairs included in the multiplex PCR systems DUP and LIN. DUP is a duplex for species identification of *Pardosa nigra* and *P. saturatior*, LIN amplifies the linyphiid spider species *Diplocephalus helleri*, *Erigone tirolensis*, *Janetschekia monodon*, *Meioneta nigripes* and *Entelecara media*. Columns show the targets and corresponding primer names (A and S denote forward and reverse primers, respectively), primer sequences in 5’-3’ direction, the size of the resulting amplicon, and the final concentration of each primer in the multiplex reaction. Except for Ent-med-A292, the primers included in LIN have not been designed in this study but are a new combination of primers designed for species identification of linyphiid spiders in glacier forelands [3].

**Multiplex**	**Target**	**Primer**	**Primer sequence (5'-3')**	**size (bp)**	**conc. (µM)**
**DUP**	P. nigra	Par-nig-S258<br>Par-nig-A257	ATTACCTCCTTCTTTATTTTTATTG<br>TATAGAATTTCCTATATGACCAACC	118	0.4
	P. saturatior	Par-sat-S257<br>Par-sat-A256	TTGGACATATAGGAAGTTCAATG<br>CTAAAACAGGTAAAGAAAGCAAC	202	0.1
**LIN**	*D. helleri*	Dip-hel-S278<br>Dip-hel-A280	CCTCCTTCTTTGTTCTTACTATTTG<br>AAGSCCCAGCCAAGTGC	151	0.6
	E. tirolensis	Eri-tir-S281<br>Eri-tir-A283	GGAGCTTGGGCTGCTATAGTA<br>AGGRACTAATCAGTTACCAAAYCCT	186	0.2
	J. monodon	Jan-mon-S282<br>Jan-mon-A284	GATATTAGGAGCTCCTGATATAGCC<br>ATAAAATTAATGGCTCCCATAATC	240	0.8
	A. nigripes	Agy-nig-S287<br>Agy-nig-A288	TCAGATATAGCGTTTCCTCGTATG<br>AGTTATACCATAGCCACGTATATTTAG	264	0.15
	E. media	Ent-med-S279<br>Ent-med-A292	GAGYTAGGTCAAGTTGGAAGCC<br>ATGCCCCTCTAACGAAGAC	298	0.15

Sample screening

The DNA concentration was determined for each ten randomly selected full-body extracts and faecal samples with a NanoDrop ND-1000 (Thermo Scientific, Waltham, USA). The measured DNA concentrations ranged between 40 and 150 ng/µl in full-body DNA extracts of *Pardosa* spp. but were below 1 ng/µl or not measurable in all faecal samples. First tests with the faecal samples indicated that this extremely low DNA concentration will not be reliably amplified under standard PCR conditions. Consequently, the amount of DNA extract added as template to the PCRs was increased from 1.5 µl to 2.7 µl and the detection threshold for the PCR products on QIAxcel was lowered from 0.1 relative fluorescent units (RFU) to 0.05 RFU, a value still above the background noise, to compensate at least partly for this low DNA concentration in faecal samples.

When the duplex PCR system (DUP) was applied in the screening of the field samples, we assumed that DNA of the consumer was amplified if only one type of DNA was present. In case DNA of both species was found, the consumer was identified as the species producing the stronger signal. This identification of the consumer was only possible because DUP was optimized to amplify both species with about the same efficacy, so that a stronger signal indicates a higher concentration of template DNA compared to the respective other species.

Reduced sensitivity has been reported for multiplex PCR systems in which the DNA of one target dominated the DNA extract [5]. To compensate the excess of predator DNA in full-body DNA extracts of lycosid spiders when screening for prey DNA with the multiplex PCR system IPC [2], the concentration of the primer pair for *Pardosa* spp. was reduced to 0.04 µM for the screening of lycosids. No such negative effect was expected for faecal samples due to the overall low DNA concentration in this sample type.

In each PCR, positive (artificial mix of DNA from the target organisms) and negative (water instead of DNA extract) controls were run along with the samples to check for correct amplification and carry-over contamination with DNA.

Samples where no DNA was detected with any of the multiplex PCR systems were re-tested with general primers C1-J-1859 [6]and HCO2198 [7] to check for the presence of amplifiable DNA. If no DNA was detected with these primers, both samples from the respective individual (full-body DNA extract and faecal sample) were excluded from the analysis.

From a total of 189 spiders, faecal samples and full-body DNA extracts were tested with different multiplex PCR systems to investigate the suitability of faecal samples for molecular analysis. In four faecal samples no amplifiable DNA was present, and these were excluded from the analysis together with the respective full-body DNA extracts, leaving 185 individuals for the comparison between the two sample types.

References

1. Juen A, Traugott M: **Detecting predation and scavenging by DNA gut-content analysis: a case study using a soil insect predator-prey system.** *Oecologia* 2005, **142:**344-352.
2. Sint D, Raso L, Traugott M: **Advances in multiplex PCR: balancing primer efficiencies and improving detection success.** *Methods Ecol Evol* 2012, **3:**898-905.
3. Raso L, Sint D, Rief A, Kaufmann R, Traugott M: **Molecular identification of adult and juvenile linyphiid and theridiid spiders in Alpine glacier foreland communities.** *PLoS ONE* 2014**:**e1017555.
4. King RA, Read DS, Traugott M, Symondson WOC: **Molecular analysis of predation: a review of best practice for DNA-based approaches.** *Mol Ecol* 2008, **17:**947-963.
5. Traugott M, Symondson WOC: **Molecular analysis of predation on parasitized hosts.** *Bull Entomol Res* 2008, **98:**223-231.
6. Simon C, Frati F, Beckenbach A, Crespi B, Liu H, Flook P: **Evolution, weighting, and phylogenetic utility of mitochondrial gene-sequences and a compilation of conserved polymerase chain-reaction primers.** *Ann Entomol Soc Am* 1994, **87:**651-701.
7. Folmer O, Black M, Hoeh W, Lutz R, Vrijenhoek R: **DNA primers for amplification of mitochondrial cytochrome c oxidase subunit I from diverse metazoan invertebrates.** *Mol Mar Biol Biotechnol* 1994, **3:**294-299.
